# Supplementary material for: Hyperdirect insula-basal-ganglia pathway and adult-like maturity of global brain responses predict inhibitory control in children
Source: Nat Commun. 2019 Oct 22;10:4798. doi: 10.1038/s41467-019-12756-8 (PMC6805945; doi:10.1038/s41467-019-12756-8)
Supplement: Supplementary file 1 — Supplementary Information [file 41467_2019_12756_MOESM1_ESM.pdf]

Supplemental Information:

Hyperdirect insula-basal-ganglia pathway and adult-like maturity of global brain responses predict inhibitory control in children

*Weidong Cai, Katherine Duberg, Aarthi Padmanabhan, Rachel Rehert, Travis Bradley ,
Victor Carrion, Vinod Menon*

I. SI Methods

Stanford_Child cohort: participants

A total of 78 fifth grade children were recruited from the East Palo Alto Ravenswood School District and South San Jose Unified School District as part of a longitudinal mindfulness training study. Only baseline data (before training) was used in the current study. Behavioral data and head motion in scanner were screened to ensure high quality of behavioral and fMRI data used in the data analysis. Behavioral and head motion criteria are explained in the behavioral analysis and fMRI preprocessing sections. Two children did not complete the scan. Thirty-eight children were excluded because of excessive head motion (N=33) and/or poor behavioral performance (N=5). The final dataset includes 38 participants (12 female, all right handed with no history of neurological or psychiatric disorders, 9-12 years of age, mean 11.03). No child was under stimulant medication. Other neuropsychological scores were summarized in **Supplementary Table S5**.

Stanford_Child cohort: behavioral paradigm and analysis

Each child performed two runs of the SST. Subjects were instructed to respond as quickly as possible to green arrows (Go Signal) by clicking with their right pointer or middle finger based on the direction of the arrow. In 25% of the trials, after a variable delay, the green arrow turned red (Stop Signal), indicating that the subject should cancel their response. The delay between the Go Signal and the Stop Signal, the SSD varied across trials in a step-wise fashion and adjusted dynamically to the subject's performance: beginning at 165ms, it decreased by 33ms for a failed stop, and increased by 33ms for a successful stop. There were 2 blocks of 96 trials each, in which 32 stop trials were randomly distributed across the block. Each Go Signal was preceded by a jittered inter-trial-interval, and a fixation cross for a duration of 500ms. For each subject, "Go Accuracy" and "Stop Accuracy" were calculated from the proportion of correct trials in Go trials and Stop trials, respectively. "Go RT" and "Failed Stop RT" were averaged reaction times (RT) in all correct Go trials and all failed Stop trials, respectively. SSRT was calculated from the distribution of Go RTs and the probability of failed Stop trials using an integration method, which is based on the Race Model [1]. Participants with less than 80% accuracy on Go trials, or with greater than 80% or less than 20% accuracy on the Stop trials, or with longer RT in unsuccessful stop trials than go trials, in either fMRI run were excluded from further analysis to ensure accurate estimation of the SSRT.

Stanford_Child cohort: MRI data acquisition

The fMRI data in the Stanford_Child cohort was collected in a single session at the Richard M Lucas Center for Imaging at Stanford University. Images were acquired on a 3T GE Signa scanner using an 8-channel head coil. Each participant was instructed to stay as still as possible during the scanning, and inflatable pillows were placed around the child's head in order to further minimize head movement. Functional images of 29 axial slices, parallel to the anterior/posterior commissure line and covering the whole brain, were acquired using a T2*-weighted gradient-echo spiral in-out pulse sequence [2] with the following parameters: slice-thickness = 4.0mm, repetition time (TR) = 2000ms, echo time (TE) = 30ms, flip angle = 80º. A high-order shimming method was used prior to data acquisition to reduce blurring and signal loss arising from field inhomogeneities. High-resolution T1-weighted images were acquired using a spoiled-gradient-recalled inversion recovery three-dimensional (3D) MRI sequence with the following parameters: TR = 8.4ms, TE = 1.8ms, flip angle = 15°, FOV = 22cm, matrix=256x192.

fMRI preprocessing

Functional MRI data from all three datasets were preprocessed using SPM8. The preprocessing pipeline included realignment, slice-timing correction, normalization to MNI space, and smoothing using a 6mm full-width half-maximum Gaussian kernel to decrease spatial noise. Maximum displacement was calculated based on parameters from realignment procedure. Subjects whose mean scan-to-scan movement were less than 0.5mm [3] and maximum displacement exceeded 5 mm [4] in either run were excluded from the analysis.

Classification analysis

We examined whether voxel-wise activation pattern within STN ROIs could differentiate between Go and SuccStop. To do so, we applied multivariate classification using the linear support vector machine algorithm (C=1) from an open-source library - LIBSVM (http://www.csie.ntu.edu.tw/~cjlin/libsvm/) as the classification tool. The contrast images of Go and SuccStop in each dataset were used as features in the classification analysis. The performance of classification was evaluated using a leave-one-subject-out cross-validation procedure. In this procedure, a pair of Go and SuccStop images from one subject was selected as a test set. The remaining participants' data was used to train a classifier, which was then applied to the test set to predict whether the images are Go or SuccStop. This procedure was repeated N times (N is the number of subjects in each dataset), with each subject's data used exactly once as a test set. The average prediction accuracy across all test sets is termed as the cross-validation accuracy. A permutation procedure was used to infer statistical significance of the cross-validation accuracy for each cluster and the accuracy difference between clusters in each dataset. Specifically, in each permutation, data labels were randomly shuffled. Cross-validation accuracies from 500 permutations were used to construct the empirical null distribution in each dataset from which p-values for cross-validation accuracies were obtained.

Task-modulated effective connectivity and behavioral correlation analysis

Task-modulated effective connectivity was computed using seed-based generalized psychophysiological interactions method (gPPI). Seeds were selectively placed in the rAI, rIFC, rMFG and rPreSMA, as we were testing a hyperdirect pathway model, which projects from cortical regions to the STN. The gPPI model consisted of a physiological variable (the raw time series of a seed), multiple psychological variables (hemodynamic response function convolved main effect of condition of interest, Go, SuccStop and UnsuccStop), and multiple interaction variables (deconvolved raw time series of the seed multiplied by main effect of condition of interest, and then convolved with the hemodynamic response function) [5]. Task-modulation effect was computed by subtracting beta values for interaction variables between task conditions (e.g. SuccStop versus UnsuccStop). We computed task-modulated effective connectivity of the STN and computed its correlation with individual's SSRT using Pearson's correlations. Multiple linear regression was used to examine whether any brain-behavior relationships could be driven by other potential confounds such as age, gender and head motion.

Replication with different head motion regressors

We conducted additional analysis to examine whether brain-behavior regression results are stable when controlling for mean rather than maximum frame-wise displacement.

Replication using volume repair analysis

We conducted additional analysis to censor scan volumes with excessive head motion using a similar procedure commonly implemented in previous developmental neuroimaging studies [6, 7]. Specifically, volumes with head motion exceeding 0.5 voxels or spikes in global signal exceeding 5% were interpolated using adjacent scans. Then, we conducted the same analyses to examine brain-behavior relation.

Replication with restricted age range in adult samples

We conducted additional analysis to examine whether a restricted age range (18-26 years old) in the adult samples impacts our findings. In the OpenfMRI_Adult1 dataset, only 11 adults are under 27 years old, which has low power in the group level analysis and no voxel survived at corrected threshold (p<0.01, FDR corrected). In the OpenfMRI_Adult2 dataset, there are 22 adults under 27 years old, which allows us to test whether our findings depends on the age range of adult samples.

Replication with additional neuropsychological measures as regressors

We conducted additional analyses to examine whether other potential confounds may explain individual differences in developmental population. We included additional regressors that measure children's cognitive development in the regression analyses, such as Sequential Processing, Simultaneous Processing, Learning Ability, Planning Ability and Mental Processing Index from Kaufman Assessment Battery for Children (KABC) [8].

Replication using SST fMRI data from the ABCD study

We analyzed SST fMRI data from the first 500 typically developing children (TDC) ages 9-11 in the ABCD study (https://abcdstudy.org/). We downloaded minimal preprocessed data from the ABCD study (two SST sessions per subject), and then normalized it to the Montreal Neurological Institute (MNI) 2mm template (91x109x91) and smoothed the resulting images using a 6mm Gaussian kernel. We then created a task design matrix using the event and onset data provided by the ABCD study and ran the same GLM and gPPI analyses as we did on the Stanford_Child, OpenfMRI_Adult1 and OpenfMRI_Adult2 datasets.
Because of missing data or event conditions, we could not complete GLM analysis on 17 participants. Among the remaining 483 participants, we excluded 240 participants because their head motion displacement was over 5mm (the criterion we used in our main analysis). We excluded individuals who had poor behavioral performance (less than 80% accuracy on Go trials, or with greater than 80% or less than 20% accuracy on Stop trials, or with longer RT in unsuccessful stop trials than go trials) and individuals who have outliers in key behavioral measure (i.e. SSRT) and brain measures (e.g. NMI, STN activation and PPI weight). Outliers was defined by more than 3 standard deviations away from the mean. The final sample included 186 TDC (9-11 years old, 104F/82M).

II. SI Notes

Similarity of brain activation patterns in children and adults during Go and Unsuccessful Stopping

We conducted additional analyses to examine spatial correlation between Child and Adult on Go and Unsuccessful stopping. We found that the spatial activation pattern on Go trials was correlated between Stanford_Child and OpenfMRI_Adult1 cohorts (r=0.36, **Supplementary Figure S1**) and between Stanford_Child and OpenfMRI_Adult2 cohorts (r=0.41, **Supplementary Figure S1**). Error-elicited spatial activation pattern was correlated between Stanford_Child and OpenfMRI_Adult1 cohorts (r=0.43, **Figure S2**) and between Stanford_Child and OpenfMRI_Adult1 cohorts (r=0.31, **Figure S2**). Overall, the correlation coefficients on Go and Unsuccessful Stop trials was much lower in comparison to spatial correlation during Successful Stop trials (r=0.69 and r=0.71, **Figure 3D**).

Examining dissociable activation patterns in adults' STN

We repeated the same analyses to detect differential STN response associated with inhibitory control in the two adult cohorts. We found significant cross-validation accuracy in the OpenfMRI_Adult2 dataset (rSTN: ACC=62.5%, p=0.04; lSTN: ACC=70.9%, p=0.004, permutation test) but not in the OpenfMRI_Adult1 dataset (rSTN: ACC=47.2%, p=0.45; lSTN: ACC=58.3%, p=0.16, permutation test).

Although STN activation was inversely correlated with SSRT in our child cohort, this relationship was not observed in the two OpenfMRI adult cohorts. Previous findings in adults regarding the relationship between STN and SSRT have also been mixed[17, 18,44,25]. Further research at higher field strengths and more precise electrophysiological studies are needed to resolve these discrepancies.

STN activation in relation to inhibitory control in adults

We repeated the same analyses to examine the relation between STN activation and SSRT in the two adult datasets. We did not find a significant correlation between activation in STN and SSRT (p's>0.05, Pearson's correlation, **Supplementary Table 11, S12**).

It is not completely clear why a significant correlation between rAI-STN connectivity and SSRT emerged in children but not in the two adult cohorts. One possibility is that children may be more dependent on Stop-signal driven modulation of the hyperdirect cortical-STN pathway because of immature proactive and anticipatory control mechanisms. Adults, on the other hand, may better implement proactive control mechanisms, which is less dependent on the hyperdirect cortical-STN pathway. Further research is required to test this hypothesis.

Cortical-STN connectivity in relation to inhibitory control in adults

We repeated the same analyses to examine whether task-modulated functional connectivity between the rAI and STN predicted SSRTs in the two adult datasets. We did not find a significant correlation between task-modulated connectivity between the rAI and STN and SSRT (p>0.05, Pearson's correlation).

Replication with different head motion regressors

Including mean, rather than max, frame-wise displacement, does not have a significant impact in the regression analyses. Multiple linear regression analyses, that included SSRT as the dependent variable and STN activation, age, gender, and mean head motion displacement as independent variables, showed that task-modulated connectivity between the rAI and rSTN was the best predictor for SSRT after controlling effect of age, gender, and head motion (p=0.006) (**Supplementary Table 13**).

Replication using volume repair analysis

Neural maturity indices in children related to individual inhibitory control abilities
We found a significant negative correlation between the NMI and SSRT using a reference map derived from the OpenfMRI_Adult1 reference map (r=-0.36, p=0.03, *Cohen's d*=0.77) (**Supplementary Figure 3**). We replicated this finding using the OpenfMRI_Adult2 reference map (r=-0.41, p<0.05, *Cohen's d*=0.89) (**Supplementary Figure 3**). To further examine whether this relationship was driven by other potential confounds, we conducted multiple linear regression with SSRT as the dependent variable and NMI, age, gender, and maximum head motion displacement as independent variables. We found that the NMI is the most robust predictor (OpenfMRI_Adult1: p<0.05; OpenfMRI_Adult2: p<0.05) (**Supplementary Table 14**).

Children's STN activation related to inhibitory action control ability
We found a negative correlation between STN activation in stopping and SSRT in the Stanford_Child cohort (lSTN: r=-0.38, p=0.02, *Cohen's d*=0.82; rSTN: r=-0.36, p=0.03, *Cohen's d*=0.77) (**Supplementary Figure 4**). To further examine whether this relationship is driven by potential confounds, we conducted multiple linear regression with SSRT as the dependent variable and STN activation, age, gender and maximum head motion displacement as independent variables. We found that both rSTN and lSTN activation was the significant predictor (ps<0.05) after controlling for effects of age, gender, and head motion (**Supplementary Table 15**).

Cortical-STN connectivity related to inhibitory control ability
We found that stop signal-modulated connectivity between the rAI and rSTN, but not the lSTN, during stopping was significantly correlated with SSRT (r=-0.45, p=0.004, *Cohen's d*=1.0) in children (**Supplementary Figure 5**), such that increased rAI-rSTN connectivity was associated with faster SSRTs. Connectivity between other prefrontal nodes and STN were not significantly correlated with SSRT (p>0.05). Multiple linear regression analyses, that included SSRT as the dependent variable and STN activation, age, gender, and maximum head motion displacement as independent variables, confirmed that task-modulated connectivity between the rAI and rSTN was the best predictor for SSRT after controlling effect of age, gender, and head motion (p=0.007) (**Supplementary Table S16**).

Replication with age-restricted adult sample

Neural maturity indices in children related to individual inhibitory control abilities
We computed children's neural maturity index (NMI) using a reference map derived from the subset (18-26 years old) of OpenfMRI_Adult2 reference map. We found a significant negative correlation between the NMI and SSRT (r=-0.43, p=0.007, *Cohen's d*=0.95) (**Supplementary Figure 6**). To further examine whether this relationship was driven by other potential confounds, we conducted multiple linear regression with SSRT as the dependent variable and NMI, age, gender, and maximum head motion displacement as independent variables. We found that the NMI is the most robust predictor of children's SSRT (p<0.01) (**Supplementary Table 17**).

Replication with additional neuropsychological measures as regressors
After adding additional neuropsychological measures from KABC as regressors, we replicated our findings: (i) the NMI remains to be the most robust predictor (**Supplementary Table 18**), (ii) the STN activation is the most robust predictor (**Supplementary Table 19**), (iii) the effective connectivity between rAI and rSTN is the most robust predictor of children's SSRT (**Supplementary Table 20**).

III. SI Figures

Supplementary Figure 1. Brain-wide activation patterns elicited by motor execution (Go trials) in children were related to activation patterns in two different cohorts of adult. Each data point represents one voxel's activation (beta contrast of Go vs. baseline) in gray matter mask. Source data are provided as a Source Data file.

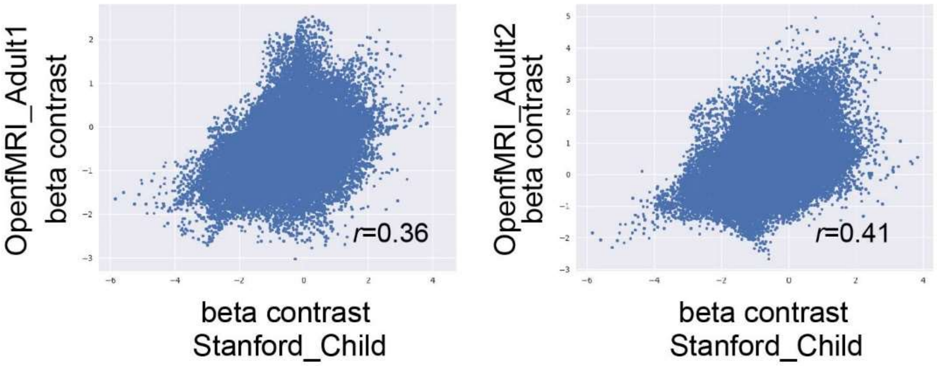

Supplementary Figure 2. Brain-wide activation patterns elicited by errors in children were related to activation patterns in the two different adult cohorts. Each data point represents brain activation in one voxel's (beta contrast of UnsuccStop versus SuccStop trials) in the gray matter mask. Source data are provided as a Source Data file.

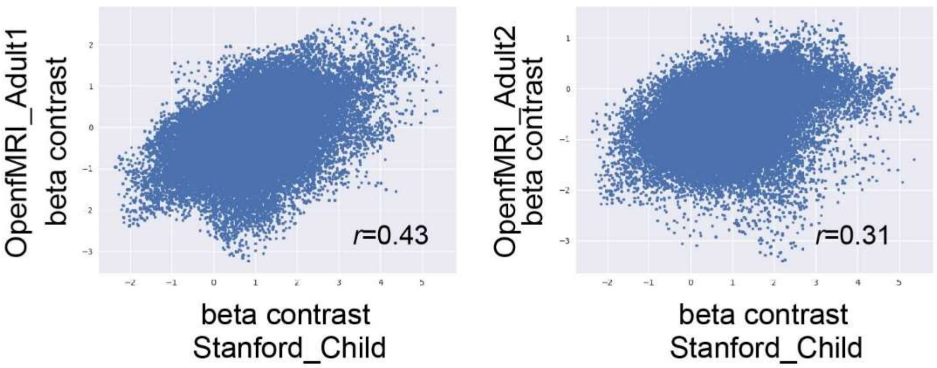

Supplementary Figure 3. NMI in children was negatively correlated with SSRT (replication after volume repair analysis). This relationship was replicated using reference maps from the two adult cohorts. Each data point represents one child. Source data are provided as a Source Data file.

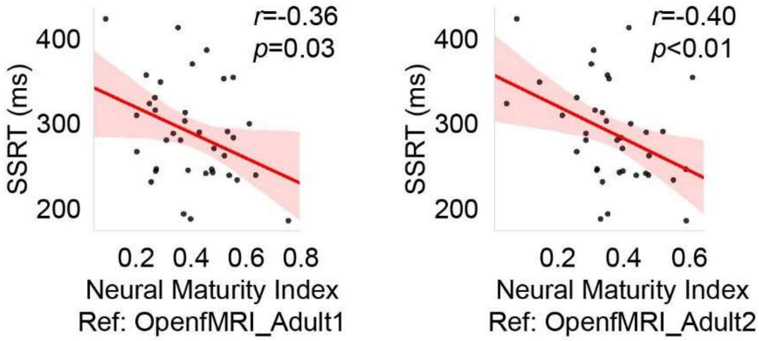

Supplementary Figure 4. STN activation during Stopping was negatively correlated with SSRT in children (replication after volume repair analysis). Each data point represents one child. Source data are provided as a Source Data file.

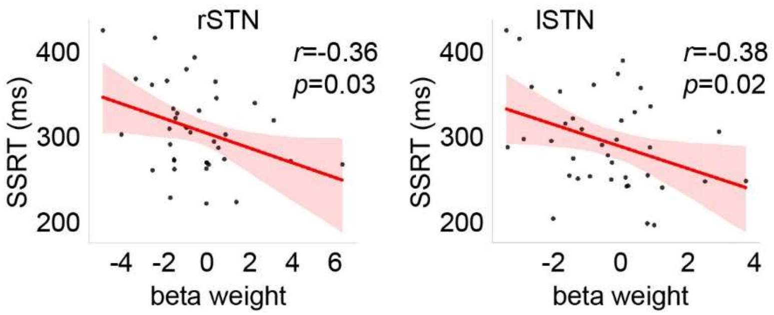

Supplementary Figure 5. Effective connectivity between the rAI (seed) and rSTN (target) was negatively correlated with SSRT in children. No such relation was observed in the left hemisphere (replication after volume repair analysis). Each data point represents one child. Source data are provided as a Source Data file.

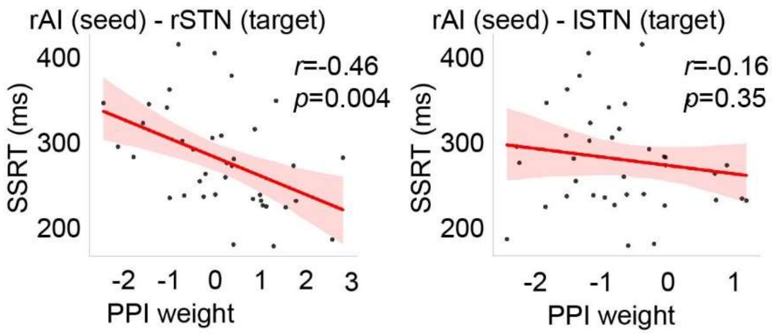

Supplementary Figure 6. NMI in children was negatively correlated with SSRT. The reference map was derived from the subset (18-26 years old) of the OpenfMRI_Adult2 cohorts. Data points represent individual children. Source data are provided as a Source Data file.

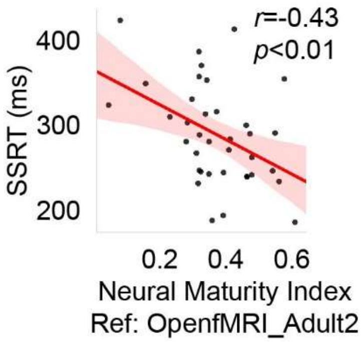

Supplementary Figure 7. NMI in children was negatively correlated with SSRT. Replication using data from the NIH-ABCD study. Each data point represents one child. Source data are provided as a Source Data file.

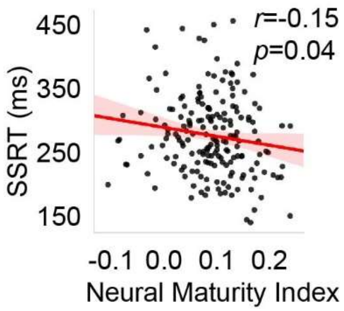

Supplementary Figure 8. STN activation during Stopping was negatively correlated with SSRT in children ages 9-11. Replication using data from the NIH-ABCD study. Each data point represents one child. Source data are provided as a Source Data file.

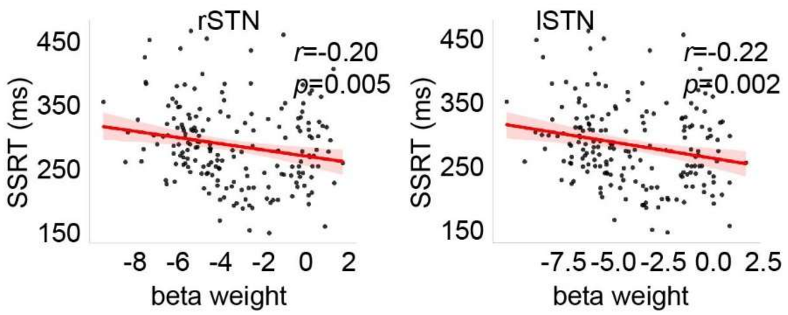

Supplementary Figure 9. Effective connectivity between the rAI (seed) and rSTN (target) was negatively correlated with SSRT in children ages 9-11. Replication using data from the NIH-ABCD study. No such relation was observed in the left hemisphere. Each data point represents one child. Source data are provided as a Source Data file.

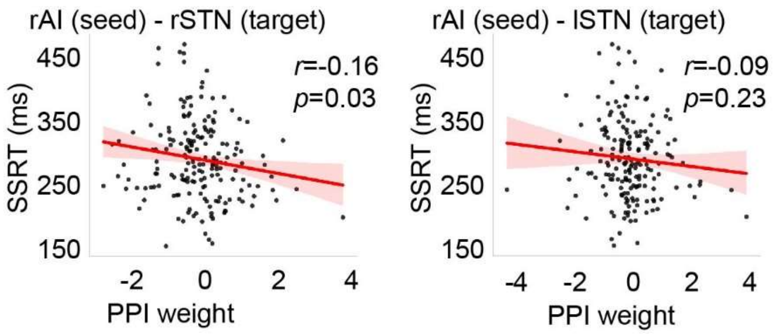

IV. SI Tables

Supplementary Table 1. Published studies and contrasts between task conditions included in meta-analysis of inhibitory control tasks in children. Most studies in children have used the Go/NoGo task.

Year	First author	Journal	Task	Contrasts	Foci (n)	Sample size*
2002	Bunge	*Neuron*	GNGT	No-go > Go	2	16
2002	Durston	*Developmental Science*	GNGT	No-go > Go	3	10
2003	Booth	*Neuroimage*	GNGT	No-go > Go	15	12
2003	Durston	*Biol Psychiatry*	GNGT	No-go > Go	7	7
2004	Schulz	*Am J Psychiatry*	GNGT	No-go > Go	5	9
2005	Booth	*J Child Psychol Psychiatry*	GNGT	No-go > Go	16	12
2006	Pliszka	*Am J Psychiatry*	SST	Correct stop > correct go	2	9
2007	Simmonds	*Neuropsychologia*	GNGT	No-go dominant foci	10	30
2008	Suskaeur	*J Cogn Neurosci*	GNGT	No-go dominant foci	7	25
2009	Bennett	*Neurotoxicol Teratol*	GNGT	No-go > Go	8	11
2012	Siniatchkin	*Brain Topography*	GNGT	No-go dominant foci	7	14
2014	Heitzeg	*Drug Alcohol Depend*	GNGT	No-go dominant foci	7	45
2015	Janssen	*Psychiatry Res*	SST	Successful inhibition > correct go	4	17
2017	van Hulst	*European Child & Adolescent Psychiatry*	GNGT	No-go > Go	13	22

* Sample size is the number of participants in the non-clinical children group in each study

Supplementary Table 2. Published studies and contrasts between task conditions included in meta-analysis of inhibitory control tasks in adults. This meta-analysis result was reported in a previous study (Cai et al., 2014).

Year	First Author	Journal	Task	Contrasts	Number of foci	Sample Size
2005	Altshuler	Biological Psychiatry	GNGT	1. NoGo > Go, Normals	4	13
2006	Aron	Journal of Neuroscience	SST	1. StopInhibit - Go<br>2. StopInhibit - StopRespond	48	5
2007	Aron	Journal of Neuroscience	SST	1. Critical StopInhibit vs. Critical Go	38	15
2004	Asahi	European Archives of Psychiatry and Clinical Neuroscience	GNGT	1. Response Inhibition	11	17
2009	Baglio	Neurobiology of Aging	GNGT	1. NoGo vs. Fixation, Healthy Controls	5	11
2004	Bellgrove	Neuropsychologia	GNGT	1. Response Inhibition	19	42
2014	Berkman	Journal of Neuroscience	SST	1. Stop>Go at T1	31	60
2010	Boecker	Human Brain Mapping	SST	1. SuccInhibit-Go	13	15
2010	Boehler	NeuroImage	SST	1. SST_SR>GT_SR<br>2. SST_SR>UST_SR	33	15
2001	Braver	Cerebral Cortex	GNGT	1. Disjunction Analysis	19	14
2009	Cai	Brain Research	SST	1. Color SS-Go<br>2. Orientation SS-Go	22	12
2011	Cai	PLOS ONE	SST	1. SST-allStop>SST-Go<br>2. SST-succStop>SST-Go<br>3. SST-succStop>SST-unsuccStop	63	26
2013	Cai	Human Brain Mapping	SST	1. Conjunction Stop-Go<br>2. Conjunction SuccStop-Go	33	23
2007	Chevrier	Human Brain Mapping	SST	1. Successful Stop-Phase Activities	3	14

2009	Chikazoe	Cerebral Cortex	GNGT	1. No-Go vs. Frequent-Go<br>2. No-Go vs. Infrequent-Go	104	25
2009	Chikazoe	Journal of Neuroscience	SST	1. Stop vs. Uncertain-Go<br>2. Stop vs. Uncertain-Go but not Uncertain-Go vs. Certain-Go	73	22
2014	Congdon	Psychiatry Research: NeuroImaging	SST	1. StopInhibit-Go, Control	4	60
2012	de Wit	The American Journal of Psychiatry	SST	1. main effect of inhibition, comparison subjects	10	37
2000	de Zubicaray	Neuropsychologia	GNGT	1. Linear Increases<br>2. Linear Increases With Number of Trials Equated Per Block	26	8
2008	Falconer	Journal of Psychiatry and Neuroscience	GNGT	1. No Go - Go, Normals	6	23
2004	Fassbender	Cognitive Brain Research	GNGT	1. Activations For Correct Inhibitions	8	21
1999	Garavan	Proceedings of the National Academy of Sciences	GNGT	1. Response Inhibition	14	14
2002	Garavan	NeuroImage	GNGT	1. Successful NoGos	16	14
2003	Garavan	NeuroImage	GNGT	1. Event-Related STOPS	7	16
2010	Hendrick	PLOS ONE	SST	1. stop as compared with go trials	18	16
2012	Hendrick	Obesity	SST	1. stop as compared with go trials for lean subjects	23	18
2004	Hester	Journal of Cognitive Neuroscience	GNGT	1. Cued and Uncued Successful Response Inhibition	21	15
2003	Horn	Neuropsychologia	GNGT	1. Go/No-Go > Go	14	21
2012	Hughes	Biological Psychiatry	SST	1. Stops>Baseline, Controls	5	10
2013	Hughes	Behav Brain Research	SST	1. Stop-inhibit>baseline<br>2. Stop-	6	15

				inhibit>Stop-respond		
2011	Jahfari	Journal of Neuroscience	SST	1. Successful stop vs. go none	7	20
2007	Kaladjian	Schizophrenia Research	GNGT	1. Correct NoGo Trials vs. Correct Go Trials, Healthy Controls	11	21
2009	Kaladjian	Bipolar Disorders	GNGT	1. No Go vs. Go, Healthy Controls, T1<br>2. No Go vs. Go, Healthy Controls, T2	20	10
2009	Kaladjian	Psychiatry Research	GNGT	1. NoGo > Go, Correct Responses, Healthy Controls	16	20
2004	Kelly	European Journal of Neuroscience	GNGT	1. Fast and Slow Successful Response Inhibitions	23	15
2000	Kiehl	Psychophysiology	GNGT	1. Task 1, Correct Rejects	8	14
1998	Konishi	European Journal of Neuroscience	GNGT	1. No-Go Dominant Foci	19	5
2007	Langenecker	Biological Psychiatry	GNGT	1. Activation in Response to Correct Rejections, Healthy Controls	8	17
2007	Leung	Journal of Neuroscience	SST	1. Conjunction Stop-Go	7	15
2006	Li	Journal of Neuroscience	SST	1. successful compared with failed inhibition	9	24
2006	Li	NeuroImage	SST	1. successful stop > failed stop, men<br>2. successful stop > failed stop, women	18	40
2001	Liddle	Human Brain Mapping	GNGT	1. Correct NoGo - Baseline<br>2. Correct NoGo - Go	42	16
2003	Maguire	NeuroImage	GNGT	1. Go/No-Go vs. Fixation<br>2. Go/No-Go vs. Go	16	6
2005	Maltby	NeuroImage	GNGT	1. Correct Inhibition, Normals	5	14

2008	Marco-Pallares	Journal of Cognitive Neuroscience	SST	1. Inhibited Trials vs. Correct Responses	10	10
2009	Mazzola-Pomietto	Journal of Psychiatric Research	GNGT	1. Significant with-group activation during response inhibition in healthy comparison subjects	7	16
2008	McNab	Neuropsychologia	GNGT	1. No-Go > Oddball (Go/No-Go)<br>2. No-Go > Go<br>3. Stop > Oddball (Stop Task)<br>4. Stop > Go	65	11
2001	Menon	Human Brain Mapping	GNGT	1. Go/NoGo - Go	13	14
2013	Montojo	Cerebral Cortex	SST	1. Successful Stopping, Controls	5	30
2003	Mostofsky	Cognitive Brain Research	GNGT	1. Primary No-Go Effects<br>2. Primary Counting No-Go Effects	6	48
2009	Padmala	Neuropsychologia	SST	1. SUCC > UNSUCC	14	35
2010	Passarotti	Psychiatry Research	SST	1. Stop > Go, Healthy Controls	5	15
2005	Ramautar	Brain Research	SST	1. SST vs. NST	7	16
2007	Roth	Biological Psychiatry	GNGT	1. Response Inhibition, Normals	13	14
2001	Rubia	NeuroImage	GNGT, SST	1. Generic Go/No-Go Activation<br>2. Generic Stop Activation<br>3. Activation Common to All Go/No-Go and Stop Task Versions	27	15
2003	Rubia	NeuroImage	SST	1. Successful Inhibition - Unsuccessful Inhibition	2	20
2006	Rubia	Human Brain Mapping	GNGT	1. Go/NoGo Task, Adults	11	23
2011	Sagaspe	NeuroImage	SST	1. (StopInhibit+StopRespond)>Go<br>2.	39	14

				StopInhibit>StopRespond		
2014	Schel	Frontiers in Human Neuroscience	SST	1. Stop Successful > Go Successful	13	24
2012	Sebastian	Psychiatry Research: NeuroImaging	GNGT, SST	1. nogo-go, control group<br>2. stop-go, control group	47	24
2010	Sharp	Proceedings of the National Academy of Sciences	SST	1. stop correct against go<br>2. stop correct against continue	16	26
2010	Simoes-Franklin	Human Brain Mapping	GNGT	1. Neutral Go-NoGo vs. Punishment Go-NoGo, Successful Inhibitions	17	16
2012	Swann	NeuroImage	SST	1. MS-SS vs. MS-Go	18	16
2012	Tabu	NeuroImage	SST	1. Stop-success vs. go in hand	15	13
2012	Townsend	Bipolar Disorders	GNGT	1. NoGo minus Go, Healthy Controls	24	30
2005	Vink	Human Brain Mapping	SST	1. Go/Stop > Go Only<br>2. Correct > Incorrect Stop	6	20
2002	Watanabe	NeuroImage	GNGT	1. Areas Activated During NO-GO Phase<br>2. Specific Activation Areas During NO-GO Phase	9	11
2009	Welander-Vatn	Bipolar Disorders	GNGT	1. Go/No-go > Fixation, Healthy Controls	12	28
2008	Xue	Cerebral Cortex	SST	1. StopInhibit-Go, Manual	13	15
2008	Zheng	Journal of Cognitive Neuroscience	GNGT, SST	1. No-Go - Go (Go/No-Go)<br>2. Stop - Go (Stop Signal)	18	18

Supplementary Table 3. Results of meta-analysis of studies involving inhibitory control tasks in adults (*p* < 0.01, FWE corrected).

				MNI coordinates		
**Cluster**	**Region**	**Volume (mm^3)**	**Extreme Value**	**x**	**y**	**z**
1	Right insula	31,240	0.089303	38	20	-4
	Right insula		0.087248	34	22	-6
	Right IFG		0.060548	48	10	28
	Right caudate		0.055126	14	8	6
	Right MFG		0.054977	42	38	20
	Right IFG		0.054297	50	16	18
	Right SFG		0.052277	28	50	30
	Right putamen		0.048693	20	8	-2
	Right MFG		0.046646	50	26	28
	Right MFG		0.043412	40	34	28
	Right MFG		0.033598	46	46	4
2	Right SMG	11,936	0.065261	52	-42	38
	Right IPL		0.051127	40	-46	46
	Right IPL		0.045121	62	-42	26
	Right IPL		0.041018	38	-56	46
	Right STG		0.040911	52	-48	14
	Right SPL		0.038902	30	-64	50
	Right precuneus		0.031374	30	-70	34
3	Right SFG	11,848	0.065965	10	14	58
	Right CG		0.062408	4	28	34
	Right MFG		0.060796	4	18	46
	Left MFG		0.045919	0	2	60
4	Left insula	8,824	0.076115	-40	16	-6
	Left insula		0.063729	-32	20	2
	Left putamen		0.047789	-14	4	6
	Left putamen		0.042635	-22	10	4
5	Right MTG	3,056	0.042222	62	-32	2
	Right STG		0.040802	54	-22	-2
	Right STG		0.040078	60	-18	-2
6	Right thalamus	2,992	0.048338	10	-10	2
	Right midbrain		0.0372	4	-20	-4
	Left midbrain		0.032972	-6	-16	-6
7	Left SMG	2,544	0.047015	-58	-48	28
	Left STG		0.030534	-58	-44	18
8	Left FG	1,104	0.045107	-40	-64	-10
9	Right MFG	1,096	0.036632	28	2	50
	Right preCG		0.027093	44	0	54
10	Left CG	1,016	0.038631	2	-24	32
11	Left IPL	992	0.031503	-48	-40	46
	Left IPL		0.029264	-42	-48	52

12	Right precuneus	496	0.029813	14	-70	52
	Right precuneus		0.029428	12	-74	44
13	Left MFG	400	0.03792	-24	-4	56
14	Left IOG	368	0.033562	-28	-94	-10
15	Right MFG	312	0.034367	24	-4	62
16	Left precuneus	264	0.029438	-28	-60	42
	Left SPL		0.028074	-26	-64	48
17	Left MFG	208	0.029143	-42	36	34

CG: cingulate gyrus; FG: fusiform gyrus; IFG: inferior frontal gyrus; IOG: inferior occipital gyrus; IPL: intraparietal lobule; MFG: middle frontal gyrus; preCG: precentral gyrus; SFG: superior frontal gyrus; SMG: supramarginal gyrus; SPL: superior parietal lobule; STG: superior temporal gyrus

Supplementary Table 4. Results of meta-analysis of studies involving inhibitory control tasks in children ($p < 0.01$, uncorrected).

				MNI coordinates		
**Cluster**	**Region**	**Volume (mm³)**	**Extreme Value**	**x**	**y**	**z**
1	Right SFG	5,136	0.014933	4	26	52
	Left SFG		0.014363	-2	16	54
	Right MFG		0.01228	4	26	42
	Left CG		0.010545	2	18	36
2	Right IFG	4,088	0.013959	46	28	8
	Right preCG		0.011464	48	12	2
	Right insula		0.009162	36	20	8
	Right IFG		0.008293	50	18	12
3	Right IFG	2,040	0.01375	54	2	24
	Right preCG		0.012059	44	8	28
4	Right SFG	1,992	0.014922	18	64	12
	Right MFG		0.008006	28	60	10
5	Left postCG	1,416	0.015586	-30	-40	64
6	Right MFG	1,408	0.015244	36	46	34
7	Left caudate	1,376	0.015091	-12	0	16
8	Right STG	1,304	0.009196	62	-36	8
	Right STG		0.00906	54	-38	14
	Right STG		0.008102	60	-44	14
9	Right IPL	1,056	0.014032	38	-30	38
10	Left hippocampus	952	0.013727	-32	-26	-12
11	Right MTG	944	0.013671	50	-13	-24
12	Left cuneus	944	0.01388	-24	-80	18
13	Left IFG	928	0.013878	-32	28	-20
14	Left insula	928	0.013808	-44	-30	0
15	Left preCG	912	0.013832	-44	4	32
16	Right MFG	912	0.013819	30	6	42
17	Left parahippo	880	0.01419	-22	-4	-24
18	Left insula	736	0.008929	-36	14	-4
19	Right cerebellum	352	0.008944	12	-54	-16
20	Right FG	352	0.009236	44	-58	-10
21	Left insula	344	0.008851	-32	24	4
22	Right MFG	344	0.008566	42	50	16
23	Right putamen	328	0.008582	26	12	-8
24	Left cuneus	328	0.008984	-18	-98	6
25	Left STG	328	0.00885	-58	-42	8
26	Right cerebellum	296	0.00878	8	-64	-10
27	Left IOG	296	0.008607	-38	-76	-4
28	Left MFG	288	0.007531	-4	40	40
29	Right hippocampus	280	0.008422	30	-26	-14
30	Right caudate	264	0.008361	18	22	-4

31	Left insula	264	0.008299	-50	8	-2
32	Left MFG	264	0.008292	-40	52	10
33	Right IPL	264	0.008298	54	-44	30
34	Right CG	248	0.007799	6	40	8
35	Right caudate	216	0.007557	4	14	2
36	Right caudate	216	0.007558	12	6	10
37	Right SFG	216	0.007573	30	52	10

CG: cingulate gyrus; FG: fusiform gyrus; IFG: inferior frontal gyrus; IOG: inferior occipital gyrus; IPL: intraparietal lobule; MFG: middle frontal gyrus; preCG: precentral gyrus; postCG: postcentral gyrus; SFG: superior frontal gyrus; SMG: supramarginal gyrus; SPL: superior parietal lobule; STG: superior temporal gyrus

Supplementary Table 5. Neuropsychological assessment and Social Economical Status scores (Stanford_Child cohort).

Kaufman Assessment Battery for Children (Standardized Scores)	
Sequential Processing	94.9 ± 12.2
Simultaneous Processing	98.0 ± 11.2
Learning Ability	108.6 ± 11.2
Planning Ability	105.5 ± 12.6
Mental Processing Index	102.2 ± 12.2
Behavior Rating Inventory of Executive Function (Normalized T-Scores)	
Inhibitory Control	52.3 ± 8.3
Shift	53.4 ± 10.8
Emotional Control	53.5 ± 11.3
Behavior Regulation	53.6 ± 10.3
Initiate	53.0 ± 11.4
Working Memory	50.8 ± 14.8
Plan/Organize	51.3 ± 14.7
Organization of Materials	48.9 ± 10.1
Monitor	50.5 ± 10.8
Metacognition Index	50.5 ± 14.6
Global Executive Composite Index	52.5 ± 10.7
BASC Self Report (Normalized T-Scores)	
Anxiety	51.6 ± 8.7
Depression	51.0 ± 8.8
Internalizing Disorders	50.7 ± 8.2
Atypicality	49.2 ± 8.4
Attention Problems	51.4 ± 12.0
Hyperactivity	47.9 ± 7.4
Social Problems	48.8 ± 8.2
Annual Household Income	
<20,000 USD	26%
20,000 – 30,000 USD	21%
30,000 – 50,000 USD	27%
Not Reported	26%

Supplementary Table 6. Brain areas activated during SuccStop vs. Go trials in children (*p* < 0.01, FDR corrected). Data from the Stanford_Child cohort.

				MNI coordinates		
**Cluster**	**Region**	**Volume (mm^3)**	**Z Value**	**x**	**y**	**z**
1	Right frontal pole	1,374	5.83	28	46	26
	Right frontal pole		3.98	50	42	28
	Right frontal pole		3.92	40	50	32
	Right frontal pole		3.75	36	38	42
2	Left SMG	319	5.82	-60	-48	32
3	Right insula	2,183	5.75	32	24	4
	Right frontal operculum		5.11	42	20	-2
	Right IFG		5.06	48	16	8
	Right preCG		4.86	50	10	32
	Right preCG		4.73	42	4	32
	Right insula		4.59	28	18	-12
4	Left insula	715	5.13	-32	18	6
	Left insula		4.73	-30	24	-2
	Left insula		4.53	-42	14	-4
5	Right SMG	1,134	4.95	50	-42	40
	Right SMG		4.44	50	-38	10
	Right SMG		4.41	54	-44	28
	Right AG		4.03	64	-46	22
	Right MTG		4	56	-50	6
	Right AG		3.78	48	-52	60
	Right SMG		3.55	52	-36	56
6	Right paracingulate	665	4.93	10	28	38
	Right SFG		4.79	6	22	52
7	Right OFG	369	4.77	26	-84	-4
	Right OFG		4.61	38	-68	-8
	Right OFG		3.97	40	-58	-6
	Right LOC		3.78	34	-82	-8
8	Right Ventricle	181	4.64	4	4	12
	Left Ventricle		4.53	-6	4	14
9	Right LOC	342	4.55	30	-66	34
	Right LOC		4.3	28	-64	48
	Right SPL		4.07	32	-56	48
	Right LOC		3.68	32	-58	58
10	Left occipital pole	650	4.51	-20	-102	4
	Left LOC		4.4	-30	-88	-6
	Left occipital pole		4.4	-24	-94	12
	Left occipital pole		4.19	-40	-92	-6
	Left LOC		4.05	-26	-88	2
	Left occipital pole		3.93	-22	-96	-2
11	Left cerebellum	17	4.51	-28	-72	-48

12	Left frontal pole	32	4.46	-26	48	42
13	Left frontal pole	313	4.36	-34	48	26
	Left frontal pole		4.31	-28	42	26
14	Right MTG	122	4.32	54	-28	-6
	Right MTG		3.43	60	-36	0
15	Right occipital pole	23	4.25	14	-104	4
	Right occipital pole		3.49	22	-102	0
16	Right SFG	56	4.11	14	8	66
	Right ITG	36	3.98	46	2	-38
17	Left MFG	44	3.8	-48	32	38
18	Right occipital pole	31	3.74	26	-90	14
	Right occipital pole		3.43	36	-92	6
19	Right MFG	43	3.72	46	8	54
	Right MFG		3.69	44	18	56
20	Right OFG	17	3.7	-34	-68	-10
21	Left preCG	10	3.57	-42	2	32
22	Left paracingulate	11	3.56	-12	24	32
23	Left OFG	11	3.54	-38	-52	-18

AG: angular gyrus; CG: cingulate gyrus; FG: fusiform gyrus; IFG: inferior frontal gyrus; IOG: inferior occipital gyrus; IPL: intraparietal lobule; ITG: inferior temporal gyrus; LOC: lateral occipital cortex; MFG: middle frontal gyrus; MTG: middle temporal gyrus; OFG: occipital fusiform gyrus; preCG: precentral gyrus; postCG: postcentral gyrus; SFG: superior frontal gyrus; SMG: supramarginal gyrus; SPL: superior parietal lobule; STG: superior temporal gyrus

Supplementary Table 7. Brain areas activated during SuccStop vs. Go trials in adults (*p* < 0.01, FDR corrected). Data from the OpenfMRI_Adult1 cohort (p<0.01, FDR corrected).

				MNI coordinates		
Cluster	Region	Volume (mm³)	Z Value	x	y	z
1	Left cerebellum	86	5.84	-28	-64	-36
	Left cerebellum		3.64	-24	-74	-32
2	Right planum polare	2,518	5.44	40	0	-20
	Right planum polare		5.18	44	-8	-14
	Right IFG		4.98	56	18	6
3	Right AG	2,938	5.32	52	-44	24
	Right STG		5.22	66	-34	4
	Right STG		5.02	52	-28	-2
4	Left planum polare	210	5.14	-46	-10	-8
	Left planum polare		4.12	-44	-2	-16
	Left planum polare		3.98	-38	-4	-22
5	Left SFG	153	5.00	-22	4	64
	Left SFG		4.12	-14	-10	64
6	Left intracalcarine	698	4.91	-18	-80	12
	Left LG		4.67	-4	-72	6
	Left occipital pole		4.56	-12	-96	14
7	Right SFG	191	4.90	10	34	46
	Right SFG		4.26	4	30	46
	Right paracingulate		3.72	10	26	32
8	Left SMG	1,211	4.84	-66	-46	8
	Left STG		4.70	-64	-30	8
	Left STG		4.66	-66	-38	14
9	Left insula	470	4.83	-36	20	8
	Left insula		4.35	-38	12	6
	Left putamen		4.34	-26	12	2
10	Right frontal pole	423	4.74	26	48	14
	Right frontal pole		4.02	50	46	20
	Right frontal pole		3.95	32	38	30
11	Left MFG	106	4.65	-32	2	60
	Left MFG		4.34	-36	4	50
12	Right SMG	143	4.57	40	-42	38
	Right AG		4.02	42	-52	44
	Right SMG		3.37	42	-44	48
13	Brain stem	23	4.49	-12	-22	-30
14	Left frontal pole	105	4.40	-30	50	10
	Left frontal pole		3.67	-36	52	2
15	Left FG	22	4.38	-38	-60	-18
16	Right putamen	36	4.36	34	-18	-8
17	Right cingulate	10	4.34	14	-2	40
18	Left MFG	99	4.33	-48	36	30
	Left frontal pole		3.83	-48	38	18

19	Right pallidum	21	4.22	24	-12	0
20	Left preCG	39	4.22	-44	4	22
21	Right thalamus	21	4.22	16	-32	-2
22	Left putamen	44	4.17	-18	6	6
	Left putamen		3.77	-20	14	4
23	Left LOC	22	4.06	-54	-66	-6
24	Right cerebellum	34	4.05	32	-48	-40
	Right cerebellum		3.92	38	-60	-38
25	Right FG	17	4.03	6	-80	-28
26	Left SMG	47	4.02	-48	-48	42
27	Right pallidum	12	4.01	12	-8	-10
28	Left FG	27	3.99	-16	-78	-26
	Left cerebellum		3.93	-6	-78	-26
29	Right frontal pole	60	3.96	46	44	2
	Right frontal pole		3.71	44	40	10
30	Left thalamus	16	3.95	-8	0	-2
31	Right putamen	36	3.91	14	12	-10
32	Left preCG	44	3.88	-24	-14	66
	Left preCG		3.88	-22	-22	66
	Left preCG		3.83	-26	-8	60
33	Right putamen	48	3.86	22	6	-4
	Right pallidum		3.72	16	6	4
	Right putamen		3.49	28	6	4
34	Left frontal pole	22	3.85	-38	56	-12
	Left frontal pole		3.54	-34	48	-10
35	Left temporal pole	10	3.81	-46	6	-10
36	Left preCG	14	3.81	-56	8	4
37	Left preCG	14	3.79	-24	-10	52
	Left SFG		3.44	-22	-2	52
38	Left MFG	15	3.77	-48	10	40
39	Left SPL	22	3.70	-30	-50	42
40	Left IFG	10	3.63	-40	16	26

AG: angular gyrus; CG: cingulate gyrus; FG: fusiform gyrus; IFG: inferior frontal gyrus; IOG: inferior occipital gyrus; IPL: intraparietal lobule; LG: lingual gyrus; LOC: lateral occipital cortex; MFG: middle frontal gyrus; preCG: precentral gyrus; postCG: postcentral gyrus; SFG: superior frontal gyrus; SMG: supramarginal gyrus; SPL: superior parietal lobule; STG: superior temporal gyrus

Supplementary Table 8. Brain areas activated during SuccStop vs. Go trials in children (*p* < 0.01, FDR corrected). Data from the OpenfMRI_Adult2 cohort (p<0.01, FDR corrected).

				MNI coordinates		
**Cluster**	**Region**	**Volume (mm^3)**	**Z Value**	**x**	**y**	**z**
1	Right insula	11,414	7.41	34	22	-4
	Right SMG		7.25	60	-40	16
	Right planum polare		6.04	48	0	-16
	Right IFG		5.94	52	20	0
	Right MTG		5.94	66	-36	2
	Right SMG		5.69	50	-42	12
	Right SMG		5.65	66	-36	26
	Right SMG		5.57	62	-36	34
	Right MTG		5.53	46	-20	-10
	Right STG		5.52	58	-30	6
2	Left planum temporale	4,918	6.63	-60	-32	16
	Left insula		5.88	-30	20	-2
	Left insula		5.82	-32	26	4
	Left planum temporale		5.62	-54	-40	18
	Left planum temporale		5.54	-40	-30	4
	Left planum temprale		5.34	-48	-30	8
	Left SMG		5.31	-62	-42	26
	Left SMG		5.23	-58	-38	46
	Left SMG		5.21	-62	-48	20
	Left Heschl's gyrus		5.18	-50	-16	0
3	Right paracingulate	2,305	5.37	10	26	40
	Right preSMA		5.27	6	4	66
	Right paracingulate		5.17	2	18	48
	Right SFG		5.11	16	12	64
	Right SFG		4.75	8	24	48
	Left paracingulate		4.35	-10	22	34
	Right cingulate		4.07	8	30	24
	Left preSMA		3.94	-2	8	62
	Left paracingulate		3.92	-6	36	34
	Left SMA		3.84	-6	6	52
4	Left cerebellum	120	5.17	-14	-78	-40
	Left cerebellum		3.34	-16	-78	-28
5	Left pallidum	492	5.08	-14	6	0
	Left putamen		4.64	-14	10	-8
	Left caudate		4.25	-14	0	16
	Left thalamus		3.95	-10	-8	2
	Left caudate		3.51	-18	16	4
6	Left frontal pole	201	4.91	-36	46	30
	Left MFG		3.75	-30	36	26
	Left MFG		3.42	-34	30	30
7	Left occipital pole	668	4.72	-24	-94	8
	Left occipital pole		4.43	-18	-98	12
	Left occipital pole		3.95	-14	-92	-6

	Left occipital pole		3.7	-8	-106	12
	Left occipital pole		3.66	-38	-92	-4
8	Right occipital pole	727	4.62	26	-92	12
	Right LOC		4.38	28	-90	-2
	Right occipital pole		4.04	16	-90	-2
	Right LOC		4	38	-84	-2
	Right occipital pole		3.76	12	-98	10
9	Left SFG	296	4.56	-12	8	72
	Left SFG		4.31	-18	6	66
10	Right cingulate	185	4.56	8	-14	32
	Right cingulate		3.93	8	-22	28
	Left cingulate		3.63	-4	-20	28
11	Left MFG	74	4.28	-48	34	30
12	Left cerebellum	11	4.24	-26	-66	-56
13	Left cerebellum	48	3.99	-32	-60	-34
	Left cerebellum		3.6	-38	-52	-34
14	Right frontal pole	16	3.94	42	48	0
15	Right precuneus	97	3.91	10	-62	46
16	Left SPL	64	3.66	-30	-48	40
17	Right hippocampus	12	3.65	24	-22	-8
18	Right cingulate	13	3.58	6	6	28
19	Left preCG	21	3.45	-44	4	20
20	Right parahippo	23	3.42	16	-28	-6
21	Left IFG	10	3.39	-60	14	18

FG: fusiform gyrus; IFG: inferior frontal gyrus; ITG: inferior temporal gyrus; LOC: lateral occipital cortex; MFG: middle frontal gyrus; MTG: middle temporal gyrus; SFG: superior frontal gyrus; SMA: supplementary motor area; SMG: supramarginal gyrus; SPL: superior parietal lobule; STG: superior temporal gyrus; preCG: precentral gyrus; preSMA: pre-Supplementary Motor Area

Supplementary Table 9. Regions of interest (ROIs) and their anatomical coordinates in MNI space.

	Coordinates (MNI)			voxel number
**ROIs**	x	y	z	
rAI	38	20	-4	123
rCau	14	8	6	123
rIFG	50	16	18	123
rMFG	50	24	28	123
rPreSMA	4	18	46	123
rSMG	52	-42	38	123
lSTN	-8	-13.5	-7	34
rSTN	11	-12.5	-7	34

Note: The rAI, rCau, rIFG, rMFG, rPreSMA and rSMA ROIs were 6mm radius spheres and their coordinates were defined by a previous meta-analytic study [9]. The lSTN and rSTN were 4mm radius spheres and their coordinates were defined by a previous ultra-high structural MRI study [10].

Supplementary Table 10. Relation between SST and cortical effective connectivity with the STN in the Stanford_Child cohort.

seed	target	r	p
rAI	lSTN	-0.03	0.87
	rSTN	**-0.46**	**0.004**
rIFG	lSTN	-0.16	0.32
	rSTN	-0.3	0.07
rPreSMA	lSTN	-0.02	0.94
	rSTN	-0.25	0.13
rMFG	lSTN	-0.24	0.16
	rSTN	-0.02	0.92

Supplementary Table 11. Relation between SST and cortical effective connectivity with the STN in the OpenfMRI_Adult1 cohort.

seed	target	r	p
rAI	lSTN	0.19	0.45
	rSTN	-0.11	0.65
rIFG	lSTN	0.01	0.99
	rSTN	-0.03	0.89
rPreSMA	lSTN	-0.07	0.78
	rSTN	-0.55	0.02
rMFG	lSTN	-0.02	0.92
	rSTN	-0.07	0.79

Supplementary Table 12. Correlation between SST and cortical effective connectivity with the STN in the OpenfMRI_Adult2 cohort.

seed	target	r	p
rAI	lSTN	0.08	0.71
	rSTN	0.23	0.3
rIFG	lSTN	-0.14	0.53
	rSTN	-0.11	0.6
rPreSMA	lSTN	-0.17	0.43
	rSTN	-0.08	0.71
rMFG	lSTN	-0.11	0.63
	rSTN	0.07	0.76

Supplementary Table 13. Multiple linear regression analysis revealed that effective connectivity between rAI and rSTN predicted children's SSRT, after controlling for age, gender, and mean framewise head motion.

	beta	*t*	*p*
Effective connectivity between rAI and rSTN during stopping	-20.3	-2.95	.006**
Age	-10.7	-0.65	0.52
Gender	2.2	0/13	0.9
Mean Frame-wise Displacement	86.8	0.61	0.55
Effective connectivity between rAI and lSTN during stopping	-3.9	-0.4	0.69
Age	-11.8	-0.63	0.53
Gender	-4.5	-0.22	0.82
Mean Frame-wise Displacement	86.76	0.53	0.6

Supplementary Table 14. Multiple linear regression analysis revealed that neural maturity index (NMI) predicts children's SSRT, after controlling for age, gender and head motion. Replication after volume repair analysis.

	beta	***t***	***p***
**Reference: Adults_OpenfMRI1**			
Neural Maturity Index	-122.1	-2.16	0.04*
Age	-11.8	-0.68	0.5
Gender	-4.84	-0.25	0.8
Maximum Frame-wise Displacement	-1.69	-0.16	0.88
Reference: Adults_OpenfMRI2			
Neural Maturity Index	-131.2	-2.52	0.02*
Age	-12.8	-0.75	0.46
Gender	-1.5	-0.08	0.94
Maximum Frame-wise Displacement	-3.1	-0.29	0.77

Supplementary Table 15. Multiple linear regression analysis revealed that activation levels in the STN predicted children's SSRT, controlling for effects of age, gender and head motion. Replication after volume repair analysis.

	beta	***t***	***p***
Activation in rSTN	-5	-2.3	.03*
Age	-12.9	-0.75	0.46
Gender	-5.6	-0.3	0.77
Max Frame-wise Displacement	-0.9	-0.09	0.93
Activation in lSTN	-5.9	-2.24	.03*
Age	-6.4	-0.37	0.72
Gender	-3.3	-0.18	0.86
Max Frame-wise Displacement	-1.6	-0.15	0.88

Supplementary Table 16. Multiple linear regression analysis revealed that effective connectivity between rAI and rSTN predicted children's SSRT after controlling for age, gender and head motion. Replication after volume repair analysis.

	beta	*t*	*p*
effective connectivity between rAI and rSTN during stopping	-18.81	-2.9	.007**
Age	-6.9	-0.4	0.68
Gender	2.4	0.13	0.9
Maximum Frame-wise Displacement	3.1	0.29	0.77
effective connectivity between rAI and lSTN during stopping	-8.6	-0.95	0.35
Age	-10.5	-0.57	0.57
Gender	-4.5	-0.23	0.82
Maximum Frame-wise Displacement	3.3	0.27	0.79

Supplementary Table 17. Multiple linear regression analysis revealed that the neural maturity index (NMI) predicts children's SSRT, after controlling for age, gender, and head motion. The reference map was derived from the subset (18-26 years old) of the OpenfMRI_Adult2 cohorts.

	beta	***t***	***p***
**Reference: Adults_OpenfMRI2 (18-26 years old)**			
Neural Maturity Index	-159.8	-2.74	0.009
Age	-13.1	-0.78	0.44
Gender	-2.2	-0.12	0.91
Maximum Frame-wise Displacement	-5.7	-0.54	0.59

Supplementary Table 18. Multiple linear regression analysis revealed that neural maturity index (NMI) predicts children's SSRT, after controlling for age, gender, and head motion, KABC sequential processing, simultaneous processing, learning ability, planning ability and mental processing index.

	beta	*t*	*p*
**Reference: Adults_OpenfMRI1**			
Neural Maturity Index	-131.7	-1.94	0.06
Age	-0.9	-0.04	0.97
Gender	-11.8	-0.51	0.62
Maximum Frame-wise Displacement	-2.0	-0.16	0.87
KABC Sequential Processing	-4.3	-0.77	0.45
KABC Simultaneous Processing	-5.6	-0.96	0.34
KABC Learning Ability	-5.9	-1.05	0.30
KABC Planning Ability	-4.3	-0.88	0.39
KABC Mental Processing Index	15.1	0.94	0.36
**Reference: Adults_OpenfMRI2**			
Neural Maturity Index	-209.7	-2.70	0.01 *
Age	-4.4	-0.22	0.83
Gender	-7.4	-0.33	0.74
Maximum Frame-wise Displacement	-4.1	-0.34	0.74
KABC Sequential Processing	-5.6	-1.07	0.29
KABC Simultaneous Processing	-7.2	-1.29	0.21
KABC Learning Ability	-7.7	-1.42	0.17
KABC Planning Ability	-5.7	-1.22	0.23
KABC Mental Processing Index	19.5	1.27	0.22

Supplementary Table 19. Multiple linear regression analysis revealed that STN activation is the most robust predictor for children's SSRT after controlling for age, gender, and head motion, KABC sequential processing, simultaneous processing, learning ability, planning ability and mental processing index.

	beta	*t*	*p*
Activaiton in rSTN	-4.7	-1.96	0.06
Age	-7.8	-0.36	0.72
Gender	-9.2	-0.40	0.69
Maximum Frame-wise Displacement	1.0	0.08	0.94
KABC Sequential Processing	-3.5	-0.64	0.53
KABC Simultaneous Processing	-4.4	-0.76	0.45
KABC Learning Ability	-4.6	-0.84	0.41
KABC Planning Ability	-3.4	-0.71	0.49
KABC Mental Processing Index	12.0	0.76	0.45
Activation in lSTN	-5.3	-1.83	0.08
Age	-0.9	-0.04	0.97
Gender	-3.6	-0.16	0.88
Maximum Frame-wise Displacement	-0.2	-0.02	0.99
KABC Sequential Processing	-3.0	-0.56	0.58
KABC Simultaneous Processing	-4.4	-0.75	0.46
KABC Learning Ability	-4.3	-0.78	0.44
KABC Planning Ability	-3.2	-0.67	0.51
KABC Mental Processing Index	11.3	0.71	0.48

Supplementary Table 20. Multiple linear regression analysis revealed that effective connectivity between rAI and rSTN predicted children's SSRT after controlling for age, gender, and head motion, KABC sequential processing, simultaneous processing, learning ability, planning ability and mental processing index.

	beta	*t*	*p*
effective connectivity between rAI and rSTN during stopping	-21.3	-2.70	0.01 *
Age	-10.6	-0.52	0.61
Gender	-0.5	-0.02	0.98
Maximum Frame-wise Displacement	3.9	0.33	0.75
KABC Sequential Processing	-2.2	-0.42	0.68
KABC Simultaneous Processing	-2.7	-0.49	0.63
KABC Learning Ability	-2.5	-0.49	0.63
KABC Planning Ability	-1.8	-0.39	0.70
KABC Mental Processing Index	6.2	0.42	0.68
effective connectivity between rAI and lSTN during stopping	-1.5	-0.12	0.90
Age	-5.7	-0.24	0.81
Gender	-9.7	-0.39	0.70
Maximum Frame-wise Displacement	1.1	0.08	0.94
KABC Sequential Processing	-2.3	-0.40	0.70
KABC Simultaneous Processing	-3.2	-0.52	0.61
KABC Learning Ability	-3.6	-0.61	0.55
KABC Planning Ability	-2.5	-0.47	0.64
KABC Mental Processing Index	8.5	0.51	0.62

Supplementary Table 21. Multiple linear regression analysis revealed that neural maturity index (NMI) predicts children's SSRT, after controlling for age, gender and head motion. Replication using data from the NIH-ABCD study.

	beta	*t*	*p*
Neural Maturity Index	-130.36	-2.02	0.04*
Age	-0.98	-1.73	0.08
Gender	1.96	0.22	0.82
Maximum Frame-wise Displacement	4.59	1.16	0.25

Supplementary Table 22. Multiple linear regression analysis revealed that activation levels in the STN predicted children's SSRT, controlling for effects of age, gender and head motion. Replication using data from the NIH-ABCD study.

	beta	*t*	*p*
Activation in rSTN	-2.98	-3.46	0.0007***
Age	-1.36	-2.4	0.02*
Gender	-4	-0.47	0.64
Max Frame-wise Displacement	5.6	1.45	0.15
Activation in lSTN	-3.22	-3.79	0.0002***
Age	-1.43	-2.52	0.01*
Gender	-4.81	-0.56	0.58
Max Frame-wise Displacement	5.53	1.44	0.15

Supplementary Table 23. Multiple linear regression analysis revealed that effective connectivity between rAI and rSTN predicted children's SSRT after controlling for age, gender and head motion. Replication using data from the NIH-ABCD study.

	beta	*t*	*p*
effective connectivity between rAI and rSTN during stopping	-9.46	-2.14	0.04*
Age	-0.93	-1.65	0.1
Gender	0.65	0.08	0.94
Maximum Frame-wise Displacement	5.02	1.28	0.2
effective connectivity between rAI and lSTN during stopping	-5.32	-1.19	0.23
Age	-0.92	-1.61	0.11
Gender	-2.08	-0.24	0.81
Maximum Frame-wise Displacement	5.14	1.29	0.19

SI Reference

1. Logan GD, Cowan WB, Davis KA. On the ability to inhibit simple and choice reaction time responses: a model and a method. *Journal of experimental psychology Human perception and performance* **10**, 276-291 (1984).

2. Glover GH. Spiral imaging in fMRI. *Neuroimage* **62**, 706-712 (2012).

3. Supekar K*, et al.* Deficits in mesolimbic reward pathway underlie social interaction impairments in children with autism. *Brain : a journal of neurology* **141**, 2795-2805 (2018).

4. Chang TT, Rosenberg-Lee M, Metcalfe AW, Chen T, Menon V. Development of common neural representations for distinct numerical problems. *Neuropsychologia* **75**, 481-495 (2015).

5. McLaren DG, Ries ML, Xu G, Johnson SC. A generalized form of context-dependent psychophysiological interactions (gPPI): a comparison to standard approaches. *Neuroimage* **61**, 1277-1286 (2012).

6. Insel C, Kastman EK, Glenn CR, Somerville LH. Development of corticostriatal connectivity constrains goal-directed behavior during adolescence. *Nature communications* **8**, (2017).

7. Iuculano T*, et al.* Cognitive tutoring induces widespread neuroplasticity and remediates brain function in children with mathematical learning disabilities. *Nature communications* **6**, 8453 (2015).

8. Kaufman AS, Kaufman NL. *Kaufman Assessment Battery for Children Second Edition*. American Guidance Service (2004).

9. Cai W, Ryali S, Chen T, Li CS, Menon V. Dissociable roles of right inferior frontal cortex and anterior insula in inhibitory control: evidence from intrinsic and task-related functional parcellation, connectivity, and response profile analyses across multiple datasets. *J Neurosci* **34**, 14652-14667 (2014).

10. Forstmann BU*, et al.* Cortico-subthalamic white matter tract strength predicts interindividual efficacy in stopping a motor response. *Neuroimage* **60**, 370-375 (2012).
