# Supplementary material for: Hyperdirect insula-basal-ganglia pathway and adult-like maturity of global brain responses predict inhibitory control in children
Source: Nat Commun. 2019 Oct 22;10:4798. doi: 10.1038/s41467-019-12756-8 (PMC6805945; doi:10.1038/s41467-019-12756-8)
Supplement: Supplementary file 3 — Reporting Summary [file 41467_2019_12756_MOESM3_ESM.pdf]

nature research

Corresponding author(s): Weidong Cai

Last updated by author(s): Mar 26, 2019

Reporting Summary

Nature Research wishes to improve the reproducibility of the work that we publish. This form provides structure for consistency and transparency in reporting. For further information on Nature Research policies, see Authors & Referees and the Editorial Policy Checklist .

Statistics

For all statistical analyses, confirm that the following items are present in the figure legend, table legend, main text, or Methods section.

n/a	Confirmed	
☐	☒	The exact sample size (*n*) for each experimental group/condition, given as a discrete number and unit of measurement
☐	☒	A statement on whether measurements were taken from distinct samples or whether the same sample was measured repeatedly
☐	☒	The statistical test(s) used AND whether they are one- or two-sided<br>*Only common tests should be described solely by name; describe more complex techniques in the Methods section.*
☐	☒	A description of all covariates tested
☐	☒	A description of any assumptions or corrections, such as tests of normality and adjustment for multiple comparisons
☐	☒	A full description of the statistical parameters including central tendency (e.g. means) or other basic estimates (e.g. regression coefficient) AND variation (e.g. standard deviation) or associated estimates of uncertainty (e.g. confidence intervals)
☐	☒	For null hypothesis testing, the test statistic (e.g. *F*, *t*, *r*) with confidence intervals, effect sizes, degrees of freedom and *P* value noted<br>*Give P values as exact values whenever suitable.*
☒	☐	For Bayesian analysis, information on the choice of priors and Markov chain Monte Carlo settings
☒	☐	For hierarchical and complex designs, identification of the appropriate level for tests and full reporting of outcomes
☐	☒	Estimates of effect sizes (e.g. Cohen's *d*, Pearson's *r*), indicating how they were calculated

Our web collection on statistics for biologists contains articles on many of the points above.

Software and code

Policy information about availability of computer code

Data collection	The fMRI data were acquired on a 3T GE Signa scanner using an 8-channel head coil.
Data analysis	FMRI data were preprocessed using SPM8. Python 2.7 was used for figure plotting and computing similarity between activation maps.

For manuscripts utilizing custom algorithms or software that are central to the research but not yet described in published literature, software must be made available to editors/reviewers. We strongly encourage code deposition in a community repository (e.g. GitHub). See the Nature Research guidelines for submitting code & software for further information.

Data

Policy information about availability of data

All manuscripts must include a data availability statement. This statement should provide the following information, where applicable:

- Accession codes, unique identifiers, or web links for publicly available datasets
- A list of figures that have associated raw data
- A description of any restrictions on data availability

Provide your data availability statement here.

Field-specific reporting

Please select the one below that is the best fit for your research. If you are not sure, read the appropriate sections before making your selection.

☒ Life sciences ☐ Behavioural & social sciences ☐ Ecological, evolutionary & environmental sciences

For a reference copy of the document with all sections, see nature.com/documents/nr-reporting-summary-flat.pdf

Life sciences study design

All studies must disclose on these points even when the disclosure is negative.

Sample size	The sample size is determined based on samples from previous neuroimaging studies in adults and children and the effect size is computed based on brain-behavior correlation analysis from previous studies using stop-signal tasks.
Data exclusions	Participants with less than 80% accuracy on Go trials, or with greater than 80% or less than 20% accuracy on the Stop trials, or with longer RT in unsuccessful stop trials than go trials, in either fMRI run were excluded from further analysis to ensure accurate estimation of the SSRT. Subjects mean scan-to-scan movement were greater than 0.5mm and/or whose maximum displacement exceeded 5 mm in either run were excluded
Replication	Two independent adults fMRI datasets from OpenfMRI.org were used to replicate the relationship between neural maturity index and behavioral performance.
Randomization	The experimental groups are children and adults. There is no randomization issue.
Blinding	blinding is not possible because the experimental groups are children and adults.

Reporting for specific materials, systems and methods

We require information from authors about some types of materials, experimental systems and methods used in many studies. Here, indicate whether each material, system or method listed is relevant to your study. If you are not sure if a list item applies to your research, read the appropriate section before selecting a response.

Materials & experimental systems

n/a	Involved in the study	
☒	☐	Antibodies
☒	☐	Eukaryotic cell lines
☒	☐	Palaeontology
☒	☐	Animals and other organisms
☐	☒	Human research participants
☒	☐	Clinical data

Methods

n/a	Involved in the study	
☒	☐	ChIP-seq
☒	☐	Flow cytometry
☐	☒	MRI-based neuroimaging

Human research participants

Policy information about studies involving human research participants

Population characteristics	The child SST fMRI dataset included 38 subjects (12 female, all right handed with no history of neurological or psychiatric disorders, 9-12 years of age).
Recruitment	Participants were recruited from San Francisco Bay area.
Ethics oversight	All datasets used in the current study were approved by their local Institutional Review Boards.

Note that full information on the approval of the study protocol must also be provided in the manuscript.

Magnetic resonance imaging

Experimental design

Design type	event-related design
Design specifications	Each participant completes two runs of the stop-signal task and each run includes 96 trials.
Behavioral performance measures	Button press and response time was recored in the experiment. The RACE model was used to compute stop-signal reaction time (SSRT).

Acquisition

Imaging type(s)	funcitonal
Field strength	3T
Sequence & imaging parameters	T2*-weighted gradient-echo spiral in-out pulse sequence with the following parameters: slice-thickness = 4.0mm, repetition time (TR) = 2000ms, echo time (TE) = 30ms, flip angle = 80º.
Area of acquisition	8-channel head coil

Diffusion MRI ☐ Used ☒ Not used

Preprocessing

Preprocessing software	SPM8
Normalization	non-linear normalization was applied on nifti format data
Normalization template	MNI152 2mm template was used.
Noise and artifact removal	head motion was regressed out
Volume censoring	visual inspection

Statistical modeling & inference

Model type and settings	random effect model was used for the 1st level stats, pearson's correlation was used for examining brain-behavior relationship.
Effect(s) tested	Successful Stop versus Go trials contrast is the main interest in the current study. No ANOVA was used.

Specify type of analysis: ☐ Whole brain ☐ ROI-based ☒ Both

Anatomical location(s)	ROIs were determined by previous meta-analysis studies and relevant high spatial resolution 7T fMRI study.
Statistic type for inference (See Eklund et al. 2016)	For brain activated map, we use p<0.01 FDR corrected.
Correction	FDR corrected

Models & analysis

n/a	Involved in the study
☐	☒ Functional and/or effective connectivity
☒	☐ Graph analysis
☐	☒ Multivariate modeling or predictive analysis

Functional and/or effective connectivity	general psychophysiological interaction
Multivariate modeling and predictive analysis	We examined whether voxel-wise activation pattern within STN ROIs could differentiate between Go and SuccStop. We applied multivariate classification using the linear support vector machine algorithm (C=1) and a leave-one-subject-out cross validation procedure. Features are beta values within STN ROIs in contrast maps.
